# Randomized double-blind, placebo-controlled study of topical diclofenac in the prevention of hand-foot syndrome in patients receiving capecitabine (the D-TORCH study)

**DOI:** 10.1186/s13063-022-06353-2

**Published:** 2022-05-19

**Authors:** Akhil Santhosh, Akash Kumar, Raja Pramanik, Ajay Gogia, Chandra Prakash Prasad, Ishaan Gupta, Nishkarsh Gupta, Winson Y. Cheung, Ravindra Mohan Pandey, Atul Sharma, Atul Batra

**Affiliations:** 1 Department of Medical Oncology, Dr BR Ambedkar Institute Rotary Cancer Hospital, All India Institute of Medical Sciences, New Delhi, 110029 India; 2 Department of Biotechnology, Indian Institute of Technology, New Delhi, India; 3 Department of Oncoanesthesia and Palliative Care, Dr BR Ambedkar Institute Rotary Cancer Hospital, All India Institute of Medical Sciences, New Delhi, India; 4 Department of Medical Oncology, Tom Baker Cancer Centre, Calgary, Canada; 5 Department of Biostatistics, All India Institute of Medical Sciences, New Delhi, India

**Keywords:** Capecitabine, Hand-foot syndrome, Diclofenac, Celecoxib, Pyridoxine, Breast cancer, Gastrointestinal cancer, Prevention

## Abstract

**Introduction:**

Hand-foot syndrome (HFS) is a common cutaneous side effect of capecitabine therapy. Apart from oral cyclooxygenase-2 (COX-2) inhibitor (celecoxib), there are no proven strategies for the prevention of HFS. However, celecoxib is associated with significant cardiotoxicity. To date, no study has evaluated the role of topical COX inhibitor, diclofenac. In this study, we aim to compare topical 1% diclofenac gel with placebo in the prevention of capecitabine-induced HFS.

**Methods:**

This is a randomized, placebo-controlled, double-blind, parallel-group superiority trial: the Diclofenac Topical in Reducing Capecitabine induced HFS (D-TORCH) study. A total of 264 patients with breast and gastrointestinal malignancies will be randomly allocated (stratified by sex and type of therapy [monotherapy or combination regimen with capecitabine]) to receive either 1% topical diclofenac or placebo that will be applied over the palmar and dorsal surface of the hands twice daily whilst taking capecitabine for 12 weeks. The patients will be followed up until the end of four cycles. The primary objective of this study is to compare the effect of topical diclofenac with placebo in preventing HFS (incidence of NCI CTCAEv5.0 grade 2 or higher HFS). The secondary objective is to compare the effect of topical diclofenac with placebo on preventing all grades of HFS (incidence of NCI CTCv5.0 all grade HFS), time to develop HFS (from the start of capecitabine), patient-reported outcomes (PROs) (HF-HRQoL questionnaire), adherence with the application (self-reported), capecitabine dose changes (number of patients with dose modifications due to HFS) and safety profile (NCICTCv5.0 all grade HFS)

**Discussion:**

The D-TORCH study aims to determine if 1% topical diclofenac reduces the incidence of grade 2 or higher HFS in patients receiving capecitabine. To date, there have been a lot of trials for hand-foot syndrome prevention using agents like pyridoxine, vitamin E, carvedilol, and various polyherbal formulations, but none has been found successful. If the trial meets the primary end point, 1% topical diclofenac will be the new standard of care for HFS prevention.

**Trial registration:**

Clinical Trials Registry of India CTRI/2021/01/030592. Prospectively registered on January 19, 2021

## Administrative information

Note: The numbers in curly brackets in this protocol refer to SPIRIT checklist item numbers. The order of the items has been modified to group similar items (see http://www.equator-network.org/reporting-guidelines/spirit-2727-statement-defining-standard-protocol-items-for-clinical-trials/).

**Table:** 

Title {1}	Randomized double-blind, placebo-controlled study of topical diclofenac in the prevention of hand-foot syndrome in patients receiving capecitabine (the D-TORCH study)
Trial registration {2}	Clinical Trials Registry of India CTRI/2021/01/030592
Protocol version {3}	Version 1.1, December 1, 2020
Funding {4}	The trial is partially funded by the Nag Foundation (non-government, not-for-profit organization. 15, Sangam Project, Phase II, 46 Dr Ambedkar Road, Pune, Maharashtra, India, 411001) Placebo and 1% topical diclofenac gel provided by Alkem Laboratories Ltd. (Devashish Building, Alkem House, Senapati Bapat Road, Lower Parel, Mumbai, Maharashtra, India, 400013)
Author details {5a}	Akhil Santhosh^1^, Akash Kumar^1^, Raja Pramanik^1^, Ajay Gogia^1^, Chandra Prakash Prasad^1^, Ishaan Gupta^2^, Nishkarsh Gupta^3^, Winson Y. Cheung^4^, Ravindra Mohan Pandey^5^, Atul Sharma^1^, Atul Batra^1^ ^1^ Department of Medical Oncology, Dr BR Ambedkar Institute Rotary Cancer Hospital, All India Institute of Medical Sciences, New Delhi, India ^2^Department of Biotechnology, Indian Institute of Technology, New Delhi, India ^3^Department of Oncoanesthesia and Palliative Care, Dr BR Ambedkar Institute Rotary Cancer Hospital, All India Institute of Medical Sciences, New Delhi, India ^4^Department of Medical Oncology, Tom Baker cancer Centre, Calgary, Canada ^5^Department of Biostatistics, All India Institute of Medical Sciences, New Delhi, India
Name and contact information for the trial sponsor {5b}	Dr Atul Batra, Associate Professor, Department of Medical Oncology Dr BR Ambedkar Institute Rotary Cancer Hospital All India Institute of Medical Sciences, New Delhi, India
Role of sponsor {5c}	This is an investigator-initiated study. The study sponsor is an academic teaching hospital and medical school, where the principal investigator is employed. The hospital provides administrative, logistic, and other supports required to conduct this study.

## Background and rationale {6a}

Capecitabine is an oral prodrug which is selectively transformed to its active moiety, 5 fluorouracil by the enzyme thymidine phosphorylase [1]. Capecitabine is commonly administered as a first and subsequent line agent in adjuvant/metastatic colorectal cancer and other gastrointestinal cancers, in patients with metastatic breast cancer who are resistant to taxane and anthracycline therapy, and pathological residual disease in patients with triple negative breast cancer following neoadjuvant chemotherapy [1–4]. Common dose-dependent toxicities of capecitabine include diarrhoea and hand-foot syndrome (HFS) [1].

HFS, also known as palmoplantar erythrodysesthesia, is characterized by painful erythema, edema of the hands and feet and varying degrees of dysesthesia, followed by skin desquamation [5]. The incidence of capecitabine-induced HFS is around 50 to 60%, whilst the severe forms account for 20–30% of cases [1]. HFS is usually self-limiting and rarely leads to severe life-threatening manifestations. In severe cases, however, it may necessitate dose interruptions, modifications, and permanent discontinuation of capecitabine [6, 7]. In addition, it may also impair the quality of life. Prior studies suggest that the peak occurrence of HFS is observed in the second cycle of capecitabine, whilst approximately 90% of HFS will be diagnosed by the fourth cycle of capecitabine [5, 8].

Several pathophysiological mechanisms of capecitabine-induced HFS have been hypothesized [9–11]. One such mechanism includes the upregulation of cyclooxygenase (COX)-2 expression [9]. Therefore, the use of COX-2 inhibitors may prevent capecitabine-induced HFS. In this respect, Lin et al. conducted a retrospective study to compare the incidence and severity of HFS in 67 patients with metastatic colorectal cancer who were subjected to capecitabine therapy with or without celecoxib and reported that the capecitabine/celecoxib group had a significantly lower rate of grade 1 HFS and a non-significant but marked reduction in grade 2 HFS as compared with the capecitabine-alone group [12]. Further, Zhang et al. performed a phase II randomized trial to assess the prophylactic efficacy of celecoxib on capecitabine-induced HFS in 110 stage II and III colorectal cancer patients and observed that celecoxib significantly reduced the incidence of grade 1 and grade 2 HFS [13]. The findings were confirmed in a phase III study by Zhang et al [14]. Despite its proven efficacy in preventing capecitabine-induced HFS, celecoxib is not widely used because of concerns for cardiotoxicity [15]. Karimi et al. reported that a polyherbal hydrogel containing *Calendula officinalis* results in fewer complications and symptoms associated with HFS in patients receiving fluoropyrimidine drugs [16]. Elif et al. reported that pyridoxine, vitamin E, emollient creams, and henna were likely to be effective in HFS symptom alleviation, but the exact doses and duration have not been ascertained [17]. Da Silva et al. noted that the release of norepinephrine from sympathetic skin terminals leads to the activation of beta 2 adrenergic receptors contributing to the upregulation of pro-inflammatory cytokines. This paves the way for use of topical agents like carvedilol, but the final results are awaited [18].

Topical non-steroidal anti-inflammatory drug inhibitors (NSAIDs), such as diclofenac, can inhibit COX-2 locally and may have a role in preventing HFS, without systemic side effects. However, no study to date has evaluated the role of topical COX inhibitors in the prevention of capecitabine-induced HFS. Therefore, this study aimed to evaluate the efficacy and safety of topical diclofenac in preventing capecitabine-induced HFS.

### Objectives {7}

We hypothesize that the application of 1% topical diclofenac will decrease the incidence of capecitabine-induced HFS (grade 2 or higher) by 15%, when compared with placebo.

The primary objective of this study is as follows:

1. To compare the effect of topical diclofenac with placebo in preventing HFS (incidence of NCI CTCAEv5.0 grade 2 or higher HFS)

The secondary objective is to compare the effect of topical diclofenac with placebo on the following:

1. Preventing all grades of HFS (incidence of NCI CTCv5.0 all grade HFS)
2. Time to develop HFS (from the start of capecitabine)
3. Patient-reported outcomes (PROs) (HF-HRQoL Questionnaire)
4. Adherence with the application (self-reported)
5. Capecitabine dose changes (number of patients with dose modifications due to HFS)
6. Safety profile (NCICTCv5.0 all grade HFS)

The tertiary correlate objectives are as follows:

1. To correlate the occurrence and severity of HFS with COX-2 levels (serum COX-2 levels)

2. To correlate the occurrence and severity of HFS with genetic polymorphism of dihydropyrimidine dehydrogenase (DPD) enzyme

The primary assessment time for the primary outcome is 4 cycles of capecitabine-based therapy, which amounts to 12 weeks.

### Trial design {8}

The Diclofenac Topical in Reducing Capecitabine induced HFS (D-TORCH) study is a single-centre, parallel-group, double-blind, 1:1 randomized placebo-controlled, superiority trial.

There was no public or patient involvement in the design of the protocol.

## Methods: participants, interventions, and outcomes

### Study setting {9}

The study will be conducted in the outpatient breast cancer and gastrointestinal cancer clinics at the Dr BR Ambedkar Institute Rotary Cancer Hospital (IRCH), New Delhi, India. The IRCH is the cancer block of the All India Institute of Medical Sciences, New Delhi, a central government-funded tertiary care hospital and academic centre. The IRCH caters to a large population (over 20 million) in Delhi, and neighbouring states, and registers over 15,000 patients with cancer annually.

### Eligibility criteria {10}

To be eligible to participate in the study, patients must meet all the following inclusion criteria:

- Age ≥ 18 years
- Starting capecitabine for breast and GI malignancies, either in adjuvant/neoadjuvant or palliative setting
- Commencing capecitabine at a dose of ≥ 1000 mg/m^2^ twice daily every 2 out of 3 weeks as single-agent chemotherapy
- Life expectancy greater than 12 weeks
- Eastern Cooperative Oncology Group (ECOG) performance status 0–2
- Able to start study treatment within 14 days of randomization
- Willing and able to comply with all study requirements, including treatment (applying diclofenac gel), timing, and/or nature of required assessments
- Signed, written informed consent

Patients meeting any of the following criteria will be excluded from the trial:

- Pre-existing grade 2 or higher neuropathy confounding assessment of HFS
- Other dermatologic conditions that in the opinion of the physician may affect the hands or feet or may complicate the evaluation during study treatment
- Concurrent or planned use of pyridoxine
- Allergic or anaphylactic reactions with NSAIDs
- History of asthma, urticaria, or other allergic-type reactions after taking aspirin or other NSAIDs
- Patients receiving oral or topical NSAIDs for other conditions
- Concurrent illnesses, including severe infections that may jeopardize the ability of the patient to undergo the procedures outlined in this protocol with reasonable safety
- Serious medical or psychiatric conditions that might limit the ability of the patient to comply with the protocol
- Pregnant or lactating women

### Who will take informed consent? {26a}

One of the study investigators will obtain a written informed consent from every patient prior to the enrolment in the clinical trial. During the consent process, the risks and benefits of participating in the study will be discussed in the language (Hindi or English) that the patient comprehends. Consent will be entirely voluntary and free from any coercion.

### Additional consent provisions for collection and use of participant data and biological specimens {26b}

Apart from the intervention, participants will be asked to provide blood samples (5 ml) at two time points: one at baseline and the second at the end of four cycles of capecitabine, or at the development of HFS (whichever is earlier). This will be used to assess serum COX-2 levels by enzyme-linked immunosorbent assay (ELISA) at two time points and genetic polymorphism of DPD enzyme in the baseline sample.

### Interventions

#### Explanation for choice of comparator arm {6b}

Patients will be randomly allocated to one of the two arms: intervention (1% topical diclofenac) or control (placebo). Placebo was chosen as the comparator arm as there is no current standard of care intervention to prevent capecitabine-induced HFS. Further, the placebo will contain the base used for topical diclofenac gel, and this will ensure that the study will elucidate purely the effect of diclofenac.

#### Intervention description {11a}

This is a double-blind study. The intervention for this trial is 1% topical diclofenac gel, whilst the placebo contains the same base used for manufacturing diclofenac gel. The tubes will be manufactured and packed in a set of standard unlabelled tubes by Alkem Laboratories. These will be labelled by the statistician using the alpha-numeric codes generated for each randomization number. Other investigators and participants will be blinded.

The participant will be provided with a pack of 16 tubes (30 gm each) to suffice for topical application over 12 weeks. The participant will apply 1 g (1 g = two fingertip units [FTU]: one FTU for one surface of one hand) of 1% topical diclofenac or placebo on the palmar and dorsal surface of each hand twice daily until discontinuation of capecitabine or 12 weeks or development of HFS, whichever is earlier. The study drug will be stored in a locked compartment at room temperature.

#### Relevant concomitant care permitted or prohibited during the trial {11d}

All patients will be allowed to receive standard of care treatment as per the physician’s discretion. Supportive care drugs including but not limited to antiemetics, anti-diarrheal, antibiotics, and bone-modifying agents such as bisphosphonates will be permitted in the study. Oral non-steroidal anti-inflammatory drugs (NSAIDs) and aspirin will not be permitted in the study, and the patients who require these drugs will need to discontinue the study treatment after discussion with the principal investigator.

#### Criteria for discontinuing or modifying allocated interventions {11b} (drop out criteria)

Patients who meet any of the following criteria will discontinue the study treatment:

- Permanent discontinuation of capecitabine by treating physician (unacceptable toxicity or progression of underlying cancer).
- Completion of 12 weeks of the study period.
- The investigator determines that the continuation of treatment is not in the patient’s best interest.
- Occurrence of an exclusion criterion affecting patient safety, e.g. pregnancy or psychiatric illness.
- Required use of a concomitant treatment that is not permitted.
- Failure to comply with the protocol, e.g. repeatedly failing to attend scheduled assessments. If a patient has failed to attend scheduled assessments in the study, the investigator must determine the reasons and document the circumstances as completely and accurately as possible in the medical records.
- The patient declines further study treatment or withdraws their consent to participate in the study.

#### Strategies to improve adherence to interventions {11c}

**Fig. 1:**
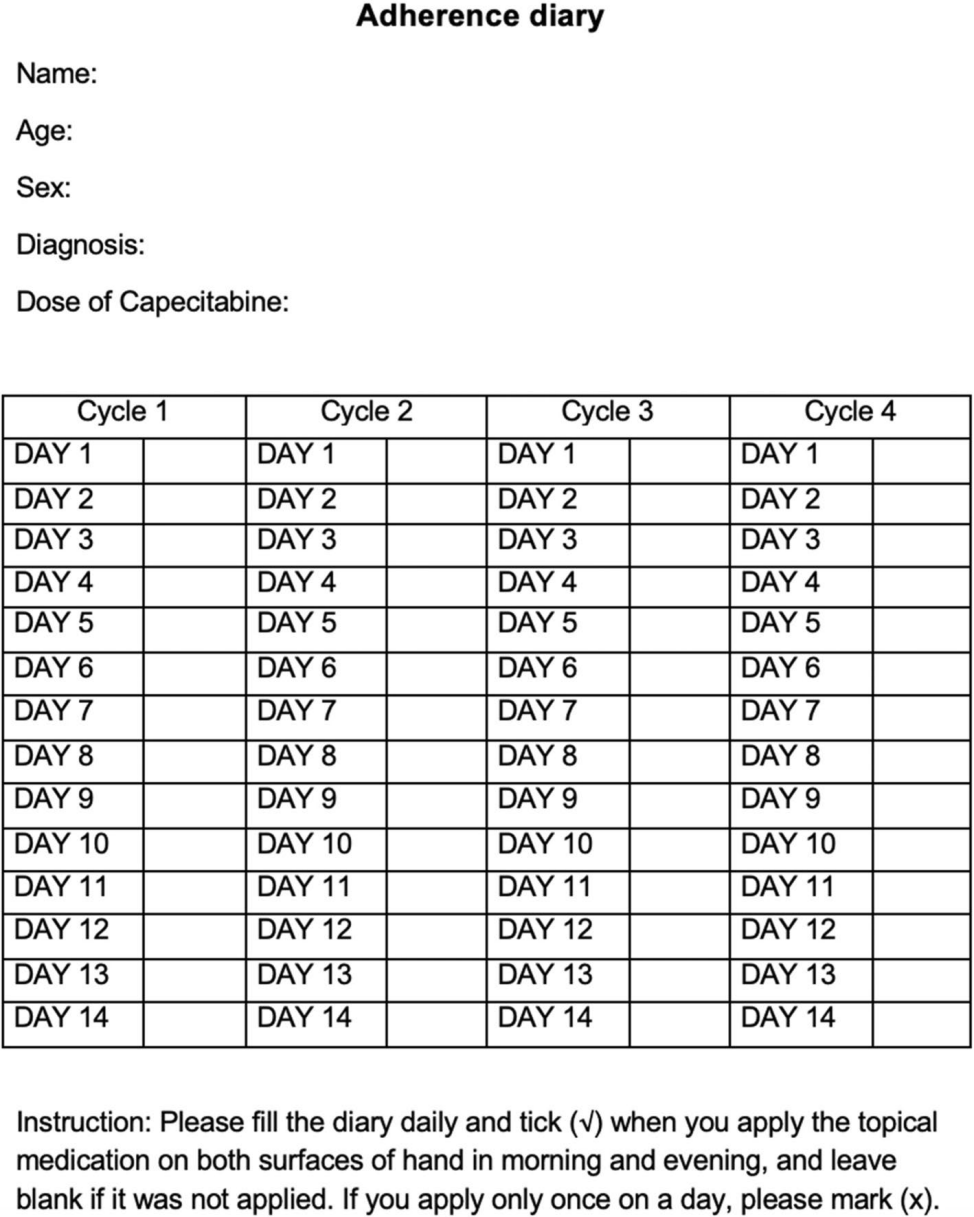
Self-reported adherence diary

Patients will be provided with a self-reported adherence diary at randomization to be filled daily (Fig. 1). This will be checked and adherence will be encouraged at each subsequent visit. Further, empty tubes will also be collected from the patient at each visit to check adherence.

#### Provisions for post-trial care {30}

Since there are no standard prophylactic interventions to prevent capecitabine-induced HFS, participants will follow the instructions provided by the treating oncologist. There is no provision to provide further topical medications after the end of the study period.

##### Ancillary and post-trial care

For prevention of hand-foot syndrome, no ancillary measures outside trial will be used. However, if the patients develop hand-foot syndrome, its treatment will be at the discretion of the treating oncologist. Likewise, post-trial care will be as per the treating oncologist.

### Outcomes {12}

The primary outcome of the study is the proportion of patients who develop grade 2 or higher HFS, as defined by the NCI CTCAE version 5.0 (Table 1).

**Table 1:** NCI CTCAEv5.0 grading for HFS/palmar plantar erythrodysesthesia

Grade	Description
**Grade 0**	No symptoms
**Grade 1**	Minimal skin changes or dermatitis (e.g. erythema, oedema, or hyperkeratosis) without pain
**Grade 2**	Skin changes (e.g. peeling, blisters, bleeding, oedema, or hyperkeratosis) with pain; limiting instrumental ADL
**Grade 3**	Severe skin changes (e.g. peeling, blisters, bleeding, oedema, or hyperkeratosis) with pain; limiting self-care ADL

*ADL* activities of daily living

The primary, secondary and tertiary outcomes of the study are described in Table 2.

**Table 2:** Primary, secondary and tertiary outcomes of the study

Outcome	Description
Primary outcomes	Grade 2 or higher HFS (proportion)
Secondary	All grades of HFS (proportion) Time to develop HFS (days) Patient-reported outcomes using HFS 14 questionnaire Adherence with topical application HFS related dose changes and delays in capecitabine Adverse events (NCI CTCAE version 5.0)
Tertiary	COX-2 levels

*HFS* hand-foot syndrome, *NCI* National Cancer Institute, *CTCAE* Common Terminology Criteria for Adverse Events, *COX-2* cycloxygenase-2

#### Participant timeline {13}

**Fig. 2:**
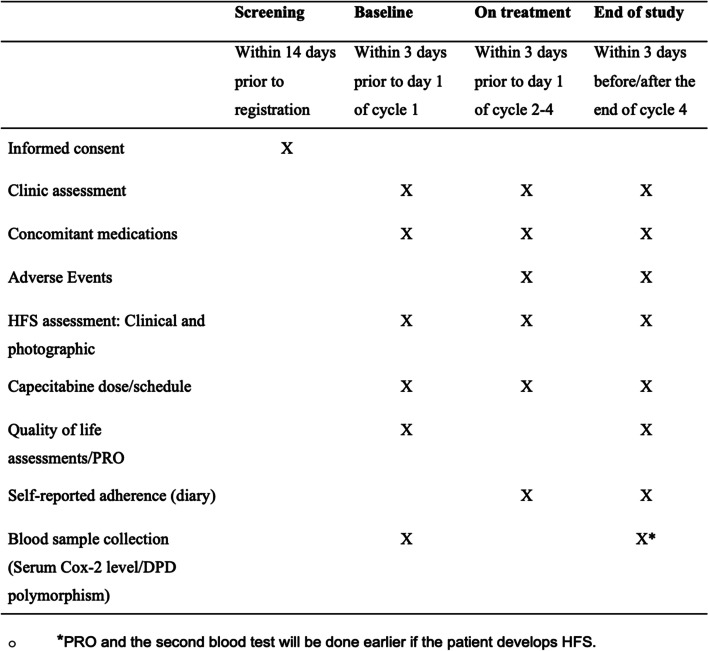
Data collection timeline (SPIRIT figure). HFS, hand-foot syndrome; PRO, patient-reported outcome; Cox-2, cycloxygenase-2

Patients will be followed up until the 21st day after the fourth cycle of capecitabine or development of grade 2 or higher HFS, whichever occurs first. Data collected for the study will include clinical characteristics and will be obtained from hospital records and routine laboratory investigations (Fig. 2). This includes baseline demographics, outcome measures, treatment details, and adverse events. Blood samples collected at the defined two time points will be labelled and stored at − 80 °C until the analysis is performed.

#### Sample size {14}

We calculated the sample size for the study using the chi-square test. With 120 patients in each study arm, there will be at least 80% power to detect a difference in HFS rates at a two-sided 0.05 significance level, using a chi-square test. This assumes that the rate of grade 2 or higher HFS is 30% in the control arm and 15% in the experimental arm. Assuming approximately 10% attrition, we plan to recruit 264 patients.

#### Recruitment {15}

Recruitment will take place in the outpatient breast and gastrointestinal cancer clinics at Dr BR Ambedkar Institute Rotary Cancer Hospital, All India Institute of Medical Sciences, New Delhi, India. Since it is a large volume teaching hospital, we expect to recruit the planned number of patients in two years.

#### Assignment of interventions

##### Sequence generation {16a}

A permuted block, computer-generated (www.sealedenvelope.com), randomization sequence with variable block size, stratified by sex (male or female), and type of therapy (capecitabine monotherapy or combination therapy) will be used to allocate participants in a 1:1 ratio.

##### Concealment mechanism {16b}

Allocation concealment will be ensured by using opaque, sealed, and sequentially numbered envelopes. The numbering of the envelopes will be conducted in advance and will be opened only after writing the participant’s name on the envelope.

##### Implementation {16c}

The allocation sequence will be generated by a person who is not involved with enrolment or future data collection and analysis. Participants will be screened and randomized at the time of planning capecitabine-based chemotherapy regimens in the outpatient clinics.

##### Blinding {17a}

The trial is a double-blind study. To ensure blinding, the statistician will label the placebo and diclofenac tubes with unique alphanumeric codes (e.g. AA1B) that are generated by the computer with the random sequence. The tubes, the packets, and the labels will look exactly the same. The study investigators and the enrolled patients will not be aware of the contents of the tubes.

##### Procedure for unblinding {17b}

The blinding will be broken by the investigator only in case of a medical emergency, where knowledge of the study drug is critical for the management of the subject’s immediate treatment. The Institute Ethics Committee will be contacted in case of unblinding for a study participant.

### Data collection and management

#### Plans for assessment and collection of outcomes {18a}

Data will be collected by the research staff at every site visit using printed copies of case record forms that will be stored in a locked compartment at the study site. All study parameters are defined a priori in a data dictionary describing data collection.

#### Plans to promote participant retention and complete follow-up {18b}

Participants will be assessed every 3 weeks and will be contacted by telephone, in case they miss their clinic appointment with the treating oncologist.

#### Data management {19}

Data entry and coding will be done manually by the research staff for the study. Data will be kept secured and stored in password-protected folders with limited access to authorized individuals.

#### Confidentiality {27}

The identifying information of study participants will be removed, wherever possible. Each study participant will be allotted a study identification number and the case record forms will contain only the de-identified data. Consent forms containing the identifying data of patients will be kept in locked compartments at the study site that can exclusively be accessed by authorized personnel.

#### Plans for collection, laboratory evaluation and storage of biological specimens for genetic or molecular analysis in this trial/future use {33}

Blood samples will be collected at pre-defined two time points, as mentioned in Fig. 2. The samples will be stored as serum and extracted DNA at − 80 °C to assess COX-2 levels and DPD polymorphism, respectively. The left-over samples will be stored at − 80 °C for future use.

## Statistical methods

### Statistical methods for primary and secondary outcomes {20a}

The primary end-point (≥ grade 2 HFS) will be compared between the two study groups by the chi-squared test, and the risk difference presented with 95% confidence intervals. The significance level will be kept at 0.05 (two-sided).

The primary analysis will be adjusted for the stratification factors used in randomization (sex and type of therapy).

Patient demographic, clinical and treatment characteristics, and other study outcomes will be described (within randomized groups and overall) using mean, standard deviation and range or median, interquartile range, and range for continuous variables and frequencies and percentages for categorical variables. Continuous variables will be checked for normality and log transformation will be conducted, where necessary. Study outcomes will be compared between the groups using standard statistical methods—two sample *t*-tests or appropriate non-parametric tests for continuous outcomes, chi-square tests for categorical outcomes, and the log-rank test for time to event outcomes. Effect sizes and 95% confidence intervals will be presented where possible. The analysis will be performed using the Stata statistical software (StataCorp. 2013. Release 13. College Station, TX) and R version 4.1.1 (R Foundation for Statistical Computing, Vienna, Austria).

### Methods for additional analyses {20b}

Subgroup analyses will be performed for sex (male vs female) and type of therapy (monotherapy vs combination therapy). Exploratory analyses for predictors of outcomes, and adjusted analyses, will use regression models including proportional hazards, logistic, and linear regression. The baseline serum Cox-2 levels will be compared with the follow-up sample by paired t-test.

### Interim analyses {21b}

No interim analysis is planned as there are minimal safety concerns with the topical application of diclofenac.

### Methods in analysis to handle protocol non-adherence and any statistical methods to handle missing data {20c}

All analyses will include all patients who were randomized and conducted using the intention-to-treat principle whereby patients are analysed according to randomized treatment regardless of treatment received. Self-reported adherence diaries will be checked and adherence will be encouraged, wherever necessary. Missing data will be checked for randomness using Little’s test [19].

### Oversight and monitoring

#### Composition of the coordinating centre and trial steering committee {5d}

The study will be coordinated by the principal investigator at the All India Institute of Medical Sciences, New Delhi. The trial steering committee will be formed by the investigators from the departments of Medical Oncology at the study site. Study coordination, monitoring, data acquisition and management, and statistical analysis will be performed by the principal investigator of the study.

#### Composition of the data monitoring committee, its role, and reporting structure {21a}

The study does not have a data monitoring committee as there are minimal safety concerns with the use of topical diclofenac. However, serious adverse events, if any, will be reviewed by the trial steering committee and reported to the Institute Ethics Sub Committee for Monitoring of Adverse Events in Clinical Trials at the site.

#### Adverse event reporting and harms {22}

Adverse events will be collected in accordance with the guidelines. Serious adverse reactions will be reported to the Institute Ethics Sub Committee for Monitoring of Adverse Events in Clinical Trials at the site.

#### Frequency and plans for auditing trial conduct {23}

After the start of the study, the trial steering committee will audit the consenting process, protocol adherence, and data collection, at six-monthly intervals. The data may be subject to audit or inspection by representatives of the Institute Ethics Committee.

#### Plans for communicating important protocol amendments to relevant parties {25}

Protocol amendments will be promptly reported to the Institute Ethics Committee and the Clinical Trials Registry of India.

#### Protocol amendments

No protocol amendment has been done till date.

#### Dissemination plans {31a}

The study will be published in a peer-reviewed journal with authorship consisting of the individual investigators and the group name ‘D-TORCH’ study investigators’. Full credit will be given to all collaborating investigators, and research staff involved in the conduct of the study. All authors will approve the manuscript prior to submission and also comply with the internationally agreed upon requirements. We will also present the results of the trial at one or more major scientific meetings. We will ensure that results are available to study participants.

#### Declaration of interests {28}

All investigators declare no financial or other competing interests.

#### Research ethics approval{24}

The study protocol was approved by the Institute Ethics Committee of All India Institute of Medical Sciences, New Delhi, on January 11, 2021 (IEC-1136/06.11.2020).

#### Authorship guidelines {31b}

The International Committee of Medical Journal Editors (ICMJE) guidelines will be followed for publishing the protocol and the final manuscript. No professional writers will be employed for drafting the manuscript.

#### Access to protocol, dataset, and statistical code {31c}

Full protocol, deidentified dataset, and statistical code will be provided as supplementary material to the final manuscript.

## Conclusions

The pathophysiology of HFS includes activation of COX-2, leading to an upregulation of the inflammatory cascade. Although systemic Cox-2 inhibitors are effective in preventing HFS, they are not used routinely because of the concerns regarding cardiotoxicity. No clinical trials have assessed the role of topical non-steroidal anti-inflammatory drugs (NSAIDs) in preventing HFS. The D-TORCH study is a randomized phase III, placebo-controlled, double-blind, parallel-group, superiority trial designed to assess the efficacy of 1% topical diclofenac in preventing capecitabine-associated HFS.

## Trial status

Recruitment of the first participant occurred on February 10, 2021, and 75 patients have been recruited by 25 August 2021. Although there was an impact on recruitment due to the second wave of COVID-19 pandemic in India, the study is progressing as expected, and it is anticipated that the last patient will be recruited by the end of September 2022, and follow-up will be completed by December 2022, thereby completing the study on time. No protocol amendments have been made to date.

## Data Availability

After the completion of the study, datasets will be made available as per the policy of the Institute Ethics Committee. There are no disclosures to be made that would restrict the availability of data.
